# Facial skin cancer surgery under local anesthesia

**DOI:** 10.25122/jml-2018-0059

**Published:** 2018

**Authors:** Anca Bordianu, Florin Bobirca

**Affiliations:** 1. “Bagdasar-Arseni” Emergency Hospital, Plastic and Reconstructive Department, University of Medicine and Pharmacy “Carol Davila” Bucharest, Romania; 2. “Dr I. Cantacuzino” Hospital, General Surgery Department, Bucharest, Romania

**Keywords:** local anesthesia, skin cancer, flap, face reconstruction

## Abstract

**Rationale:** In the last decade, the incidence of skin cancers has been increasing. Early diagnosis, treatment and prevention are crucial in helping to diminish the incidence, mortality and morbidity associated with skin cancers.

**Objective:** This article presents arguments for and against local anesthesia in the treatment of skin cancers, including the clinical cases, a summary of treatment, and prognosis.

**Methods and results:** Under local anesthesia, local and loco-regional flaps offer an optimal shape and volume for face reconstruction, minimizing the operative time and therefore the hospitalization. Facial skin cancer surgery under local anesthesia also contributes significantly to decreasing health care costs compared to general anesthesia.

**Discussions:** Although in our practice, excision of skin tumors in the facial area under local anesthesia is a frequent and harmless surgical method, it can cause increased stress in some patients. However, the benefits are significantly greater than the disadvantages.

## Introduction

Facial reconstruction after skin cancer removal is challenging and has multiple implications because, beyond the achievement of functional restoration, the surgeon must strive to provide an aesthetic outcome [1, 2].

In this regard, knowledge of tissue biomechanics is imperative for proper wound closure [3]. Tissue mobility is determined by its biology [4], a fact well known from the skin tension lines of the face, which results in easier one direction closures, but much more difficult perpendicular closures.

Apart from the above-mentioned, the success rate of surgery depends on a number of biological factors, independent of the surgeon’s abilities, such as: age, general health, medication, smoking habits, and other variables, such as: different skin characteristics (e.g., elasticity, actinic skin, sebaceous damage, skin thickness), previous scars, individual cicatrization capacity and so forth [5–7].

The main principles aimed at providing optimal closure are: (1) skin closure under minimal tension, (2) replacing “like with like”, (3) preserving major anatomical structures (e.g., lip, nose, eyebrow), and (4) scar location corresponding to functional and aesthetic units of the face [8].

The target of all facial surgical procedures is to provide complete restoration and invisible scars, even if perfection, while it is desired, is not completely achieved [9].

This article presents the arguments for and against local anesthesia in the treatment of skin cancers, including the clinical cases, a summary of treatment, and prognosis.

## Methods and materials

The study took place from 2015 to 2018. Fifty-two patients with facial tumors, with a mean age of 62.5 years, were included in the study. All patients underwent skin cancer removal, followed by reconstruction with local or loco-regional flaps, under local anesthesia.

For these cases, we used direct closure (6 cases), rotation flaps (8 cases), advancement flaps (12 cases), transposition flaps (7 cases), island pedicle flaps (11 cases), and staged pedicle flaps (8 cases).

**Figure 1:**
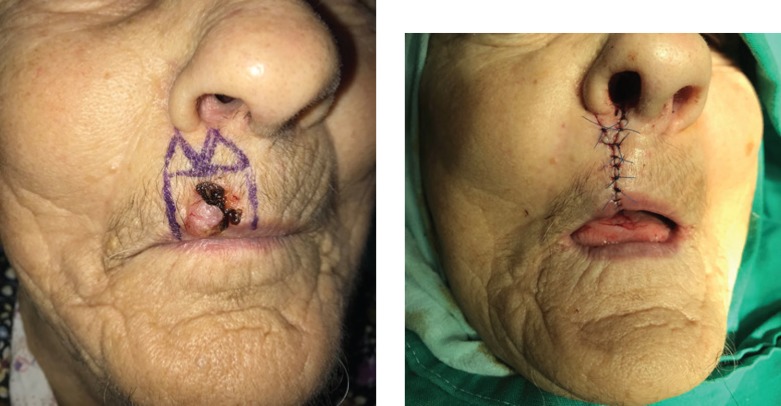
Primary closure

Direct closure (Figure 1) was used when defect size was relatively small, and the patient’s skin provided proper elasticity and volume.

Rotation flaps are frequently used when tensions from a potential primary closure would be too high. We mostly chose this type of flap in case of large defects, as tissue laxity allows rotation into the recipient site. Even if the flap is relatively large, a good design offers minimal visibility of scars. Redundant tissue may occur, and the overlapped flap tissue must be removed.

**Figure 2:**
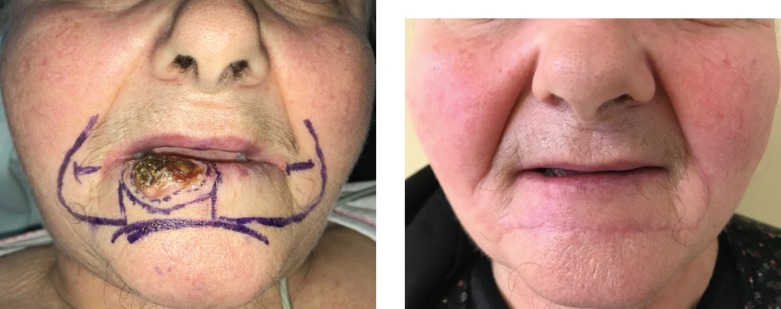
Karapandzic flap

**Figure 3:**
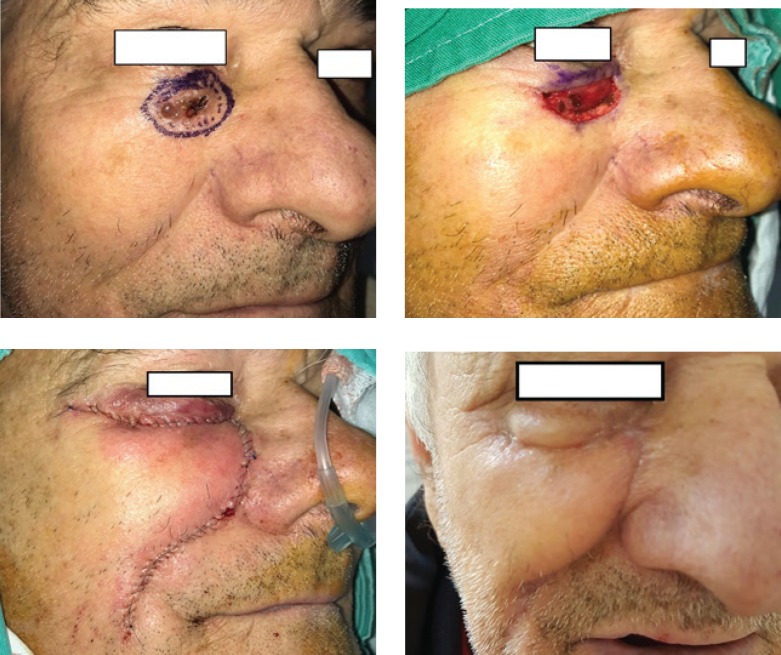
Combination of advancement and rotation flap

**Figure 4:**
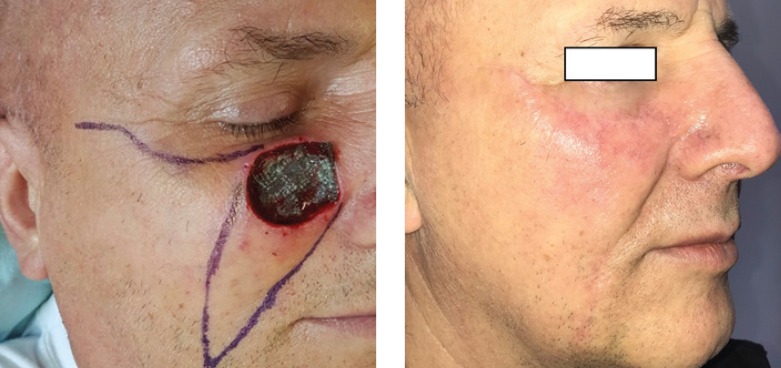
Combination of advancement and rotation flap

Advancement flaps, a common and straightforward reconstructive procedure of facial operative wounds, provides a certain tension release, and no tension redistribution. Standing tissue cones (“dog-ears”) do occur, but removal is not always necessary. This type of flap has good results on a rather flat surface, in order to obtain an aesthetic outcome, and maintain the natural contour of the face. Depending on the location of the defect, a combination of advancement and rotation flaps (Figures 2, 3 and 4) can be used.

**Figure 5:**
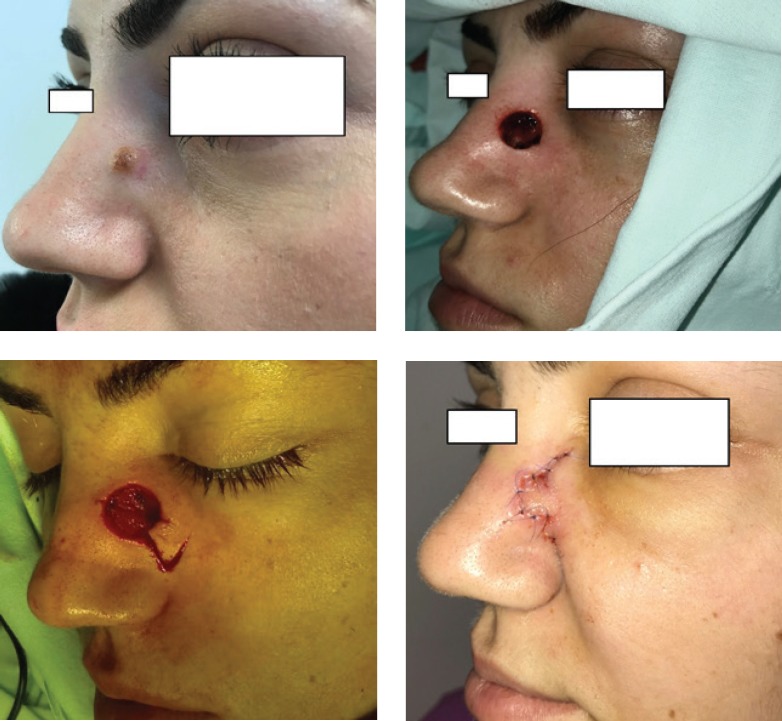
Rhombic flap

Transposition flaps, as the name suggests, transpose tissue and skin from a laxity area into the operative wound, redirecting and redistributing tension. The most commonly used design is the rhombic flap [10] (Figure 5), but we also used other types, such as the bilobed [11], trilobed, and banner flap. This type of flap offers good redistribution of the tension lines.

**Figure 6:**
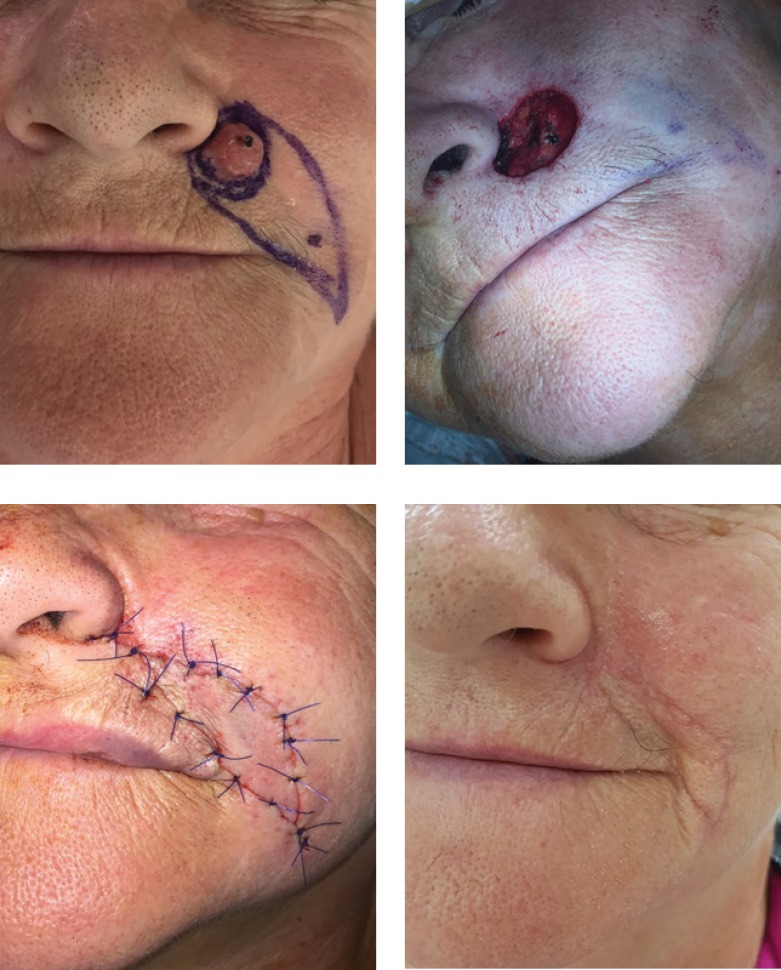
Island pedicle flap

The island pedicle flap (Figure 6) is a very movable type of flap, isolating an island of skin on a pedicle (e.g., vascularized deep fatty pedicle, deep muscular pedicle, perforator from an axial vascular bundle). Good knowledge of facial anatomy is required, in order to successfully elevate this type of flap [12]. Pincushioning is a major risk of the procedure if the flap is oversized and not properly trimmed. Also, substantial wound contraction could occur; this must be managed with a good inset of the flap.

**Figure 7:**
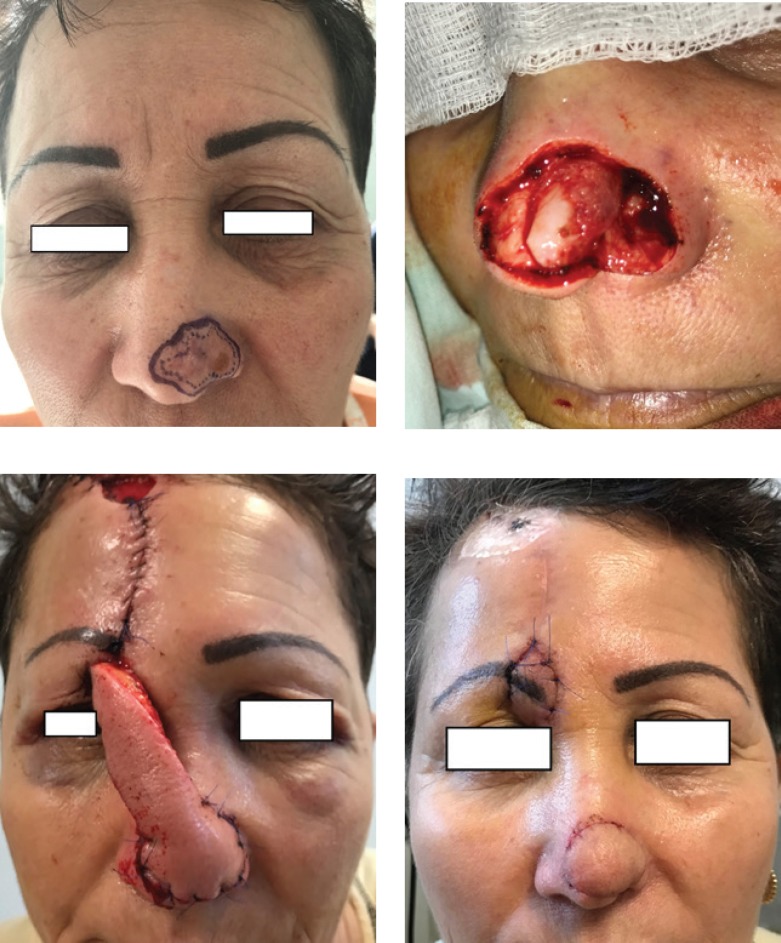
Staged pedicle forehead flap

Staged pedicle flaps (Figure 7) are mostly used when no local resources are available. A pedicle flap is transposed into the defect, and left for a determined period of time, after which the pedicle is severed. These flaps are indicated for nose [13], lip, and ear reconstructions. The main disadvantages are the initial deformed appearance, and the need for at least a second intervention, for flap revision.

In all cases, possible complications might occur [14]. The surgeon should not perform any procedure if the potential adverse consequences cannot be handled. The most common complications are: edema, hematoma, flap necrosis, which leads to flap failure, hypertrophic or depressed scars, which require revision.

## Results

All flaps survived, and the outcome was aesthetic in all cases.

Five patients had previous skin cancer surgery, which limited the laxity and volume of tissue that could be used.

No patient had major complications. Minor complications included hematoma (n=1), and edema (n=1).

The mean follow-up period was 1 year, and no recurrence was identified. For all patients, the aesthetic outcome was satisfactory.

## Discussions

Local anesthesia has a fast onset, adequate duration, with minimal systemic and local toxicity [15, 16]. The patient remains awake and cooperative during the whole procedure.

In order to obtain an effective and efficient surgical procedure [17], it is essential to have good control of pain, and hemorrhage. This also leads to prolonged post-surgical pain control.

The main advantages of using local anesthesia are the low impact on the patient’s general health, low complication and morbidity rates, patient safety, and significant cost reductions.

By using local anesthesia, patients with a poor health condition (e.g., elderly patients, patients suffering from chronic diseases), or pregnant women can be easily and safely managed.

Local anesthesia is an easy-to-apply method that requires minimal equipment and materials [18]. No additional specialized personnel is needed.

Patients must be mentally prepared for the procedure, which can be obtained through good communication between the surgeon and the patient. Also, allergic reactions to anesthetics must be taken into consideration.

However, local anesthesia is relatively contraindicated for infants, small children, mentally retarded patients, patients with significant medical diseases, or patients who are not mentally prepared for the procedure. Also, if a suppurative infection at the needle insertion site is present, the procedure must be avoided.

## Conclusions

Under local anesthesia, local and loco-regional flaps offer an optimal shape and volume for face reconstruction, minimizing risks for the patient, the operative time, thus the hospitalization.

Surgery for facial skin cancer under local anesthesia also contributes significantly lowering health care costs compared to general anesthesia.

Although in our practice, excision of facial region skin tumors under local anesthesia is a frequent and inoffensive surgery method, it can cause increased stress in some patients. However, the benefits are significantly greater than the disadvantages.

## Conflict of Interest

The authors confirm that there are no conflicts of interest.
